# Oman Vision 2040: A Transformative Blueprint for a Leading Healthcare System with International Standards

**DOI:** 10.3390/healthcare13222911

**Published:** 2025-11-14

**Authors:** Mohammed Al Ghafari, Badar Al Alawi, Idris Aal Jumaa, Salah Al Awaidy

**Affiliations:** 1 Oman Vision 2040 Implementation Follow-Up Office, Ministry of Health, Muscat, Oman; mohammed.alghafari@moh.gov.om (M.A.G.); badar.a@omsb.org (B.A.A.); 2 Directorate General of Health Services and Programs, Primary Health Care Supportive Services, Ministry of Health, Muscat, Oman; idris.aljumah@gmail.com; 3 Freelance Public Health Consultant, Muscat, Oman

**Keywords:** Oman Vision 2040, transformative blueprint, healthcare system

## Abstract

**Background/Objectives:** Oman Vision 2040, the national blueprint for socio-economic transformation, aims to elevate the Sultanate to developed nation status, with the “Health” priority committed to building a “Leading Healthcare System with International Standards” via a Health in All Policies (HiAP) approach. This paper critically reviews Oman’s strategic health directions and implementation frameworks under Vision 2040, assessing their alignment with global Sustainable Development Goals (SDGs) and serving as a case model for health system transformation. **Methods:** This study employs a critical narrative synthesis based on a comprehensive literature search that included academic, official government reports, and international organization sources. The analysis is guided by the World Health Organization’s (WHO) Health Systems Framework, providing a structured interpretation of progress across its six building blocks. **Results:** Key interventions implemented include integrated governance (e.g., Committee for Managing and Regulating Healthcare), diversified health financing (e.g., public private partnership (PPPs), Health Endowment Foundation), and strategic digital transformation (e.g., Al-Shifa system, AI diagnostics). Performance metrics show progress, with a rise in the Legatum Prosperity Index ranking and an increase in the Community Satisfaction Rate. However, critical challenges persist, including resistance to change during governance restructuring, cybersecurity risks from digital adoption, and system fragmentation that complicates a unified Non-Communicable Disease (NCD) response. **Conclusions:** Oman’s integrated approach, emphasizing decentralization, quality improvement, and investment in preventive health and human capital, positions it for sustained progress. The transformation offers generalizable insights. Successfully realizing Vision 2040 demands rigorous, evidence-informed policymaking to effectively address equity implications and optimize resource allocation.

## 1. Introduction

**Figure 1:**
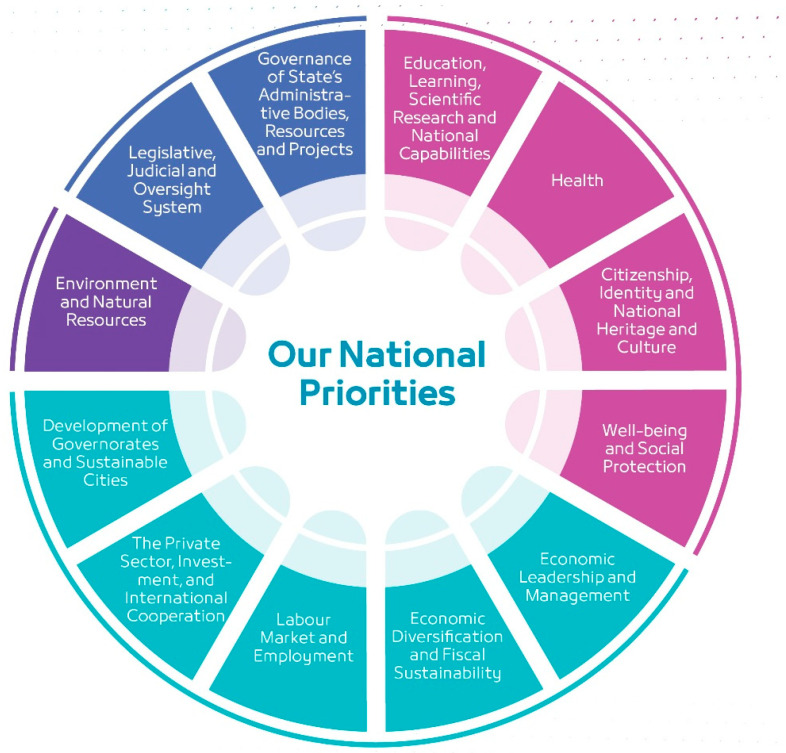
Oman Vision 2040 National Priorities. The “People and Society” pillar, encompassing Education, Health, Citizenship, and Well-being, is foundational to the vision, underscoring the integrated and cross-sectoral approach to national development. Source: Reprinted/adapted from Ref. [1].

Oman has undertaken a significant developmental journey since 1970, with Oman Vision 2040 serving as the national reference for economic and social planning from 2021 to 2040 [1]. This overarching ambition is to elevate the Sultanate to the ranks of developed countries across economic, social, environmental, and governance domains. This vision is structured around four foundational pillars: People and Society, Economy and Development, Sustainable Environment, and Governance and Institutional Performance, with health designated as a top priority under the “People and Society” pillar, shown in Figure 1 [2].

This strategic focus builds upon a robust history of public health achievements. Prior to the modern Omani Renaissance in 1970, healthcare infrastructure was rudimentary, with only two hospitals operational, which contributed to a low average life expectancy of 49.3 years in 1970 [3,4]. The establishment of the Ministry of Health (MoH) in 1970 marked a transformative period, prioritizing primary healthcare and a commitment to universal services for all citizens. These efforts resulted in notable public health outcomes, including a significant reduction in under-five mortality rates and near-universal immunization coverage [5,6].

Concurrently, Oman is navigating a significant demographic and epidemiological transition. The population has experienced a dramatic increase in life expectancy, rising to 77.2 years by 2024, and projections indicate a six-fold increase in the elderly population by 2030 [7,8,9,10]. This demographic shift, coupled with the successful control of communicable diseases, has led to a “double burden” of disease, where chronic NCDs now pose the primary public health challenge. The strategic direction of Vision 2040—“A Leading Healthcare System with International Standards”—is a direct response to these evolving challenges and is supported by five interconnected objectives aligned with the WHO Health Systems Framework [2,11].

This paper provides a critical narrative review and analysis of Oman’s strategic directions, initiatives, and implementation frameworks in health under Vision 2040. While previous research has documented Oman’s historical public health successes and analyzed specific challenges like the NCD burden, a critical knowledge gap exists in the literature regarding the system-wide, interconnected performance of the new policy agenda. Specifically, there is no comprehensive evaluation that simultaneously synthesizes progress, challenges, and implementation trade-offs across all six WHO Health Systems Framework building blocks during the Vision’s initial phase. By providing this holistic, structured, and critical analysis of the integrated strategy, this study offers a unique lens to understand how proactive policy interventions, robust governance, and strategic investments are driving a national health agenda. Crucially, it serves as a generalizable case model for other nations navigating similar complex, whole-of-system transformations.

## 2. Methodology

This review employs a critical narrative synthesis based on a comprehensive and structured literature search to ensure transparency and rigor. The analysis is guided by the World Health Organization’s (WHO) Health Systems Framework as its conceptual model [11]. This framework provides a structured approach to assessing health systems through six interconnected building blocks: service delivery, health workforce, health information systems, access to essential medicines, health financing, and leadership and governance. By applying this framework, our analysis moves beyond a descriptive overview to provide a structured interpretation of the strategic directions, initiatives, and challenges of Oman’s Vision 2040 health agenda.

### 2.1. Literature Search Strategy and Data Sources

A comprehensive search was conducted across multiple platforms to identify relevant literature and official documentation related to Oman Vision 2040 and its health sector transformation. The search strategy employed a combination of keywords related to the country, the policy framework, and health system components. The primary data sources systematically searched included:

1. Academic Databases: PubMed/MEDLINE, Scopus, and Google Scholar.
2. Official Government Publications: Oman Vision 2040 official documents, annual reports from the Oman Vision 2040 Implementation Follow-up Unit, and MoH reports.
3. International Organization Reports: Publications from the World Health Organization (WHO), World Bank, UNICEF, and United Nations Development Programme (UNDP).

The search terms used included a variety of combinations designed to capture all relevant policy, implementation, and analytical discussions, such as: “Oman Vision 2040”; “Oman health system” OR “healthcare transformation Oman”; “non-communicable diseases Oman” OR “health policy Oman”; “universal health coverage Oman”; “digital health Oman” OR “health workforce Oman”; and “GCC healthcare reforms.”

The following criteria guided the selection of sources:

Inclusion: Documents and articles published from 2010 onwards were included to cover the preceding policy context and the entire Vision 2040 period (2021 onwards), written in English, focusing on policy, strategy, performance, and analysis of Oman’s health sector. Priority was given to official reports and peer-reviewed studies.

Exclusion: Opinion pieces, editorials without empirical data, conference abstracts, and documents not directly related to Oman’s health system or Vision 2040 were excluded.

### 2.2. Selection Process and Data Extraction

The identification, screening, and inclusion of sources were conducted to ensure explicit reporting of the literature retrieval process, as summarized in Table 1. The initial retrieval identified 500 records. After screening titles and abstracts and assessing full-text eligibility, 30 sources were ultimately included in the final narrative synthesis.

Initial screening of titles and abstracts, followed by a full-text review of relevant articles, was performed independently by the first two authors. The initial consensus rate for inclusion was high, and all included sources were ultimately agreed upon by the reviewers. Key findings, policy details, and performance metrics were systematically extracted and mapped against the six WHO building blocks using a structured framework for consistent synthesis.

### 2.3. Critical Appraisal and Synthesis Strategy

Given the reliance on official policy and institutional reports, a critical appraisal was applied to ensure the reliability and objectivity of the evidence. We explicitly acknowledge the potential for institutional self-reporting bias inherent in government and WHO documents. To mitigate this risk and ensure a balanced perspective, the narrative review systematically contrasts official performance indicators with independent third-party evaluations (e.g., the Legatum Prosperity Index, World Bank/UNICEF efficiency reports). It also integrates peer-reviewed academic literature to challenge or validate claims made in policy documents, and highlights implementation challenges and trade-offs reported by researchers and external reviews, rather than solely focusing on reported achievements.

Data relevant to the review’s core themes—including policy, governance, infrastructure, human resources, financing, digital health, and SDG alignment—were systematically extracted from included sources. Findings were synthesized narratively, integrating diverse perspectives to identify trends, achievements, challenges, and policy implications, thereby providing a comprehensive and critical analysis of Oman’s health system transformation within the Vision 2040 framework.

## 3. Oman’s Health System: Analysis of Progress and Challenges

The analysis of Oman’s health system under Vision 2040 reveals a strategic effort to build on past achievements while confronting complex, modern-day challenges. This section provides a critical analysis of the country’s progress, to interpret how strategic goals are being translated into policy interventions and what challenges remain.

### 3.1. Strategic Vision and Conceptual Framework

The strategic direction for health in Vision 2040 outlines the aspiration for a ‘Leading Healthcare System with International Standards’. This vision is pursued through five key objectives that directly align with the WHO’s six health system building blocks [2,11]:

1. **Sustainable Health and Well-being of the Entire Population with Shared Responsibility by Everyone, Fostering a Healthy Society Safeguarded from Health Risks, where “Health is the Responsibility of All”:** This aligns with the leadership and governance component of the WHO framework and is reinforced by the National Health Policy’s explicit adoption of HiAP approach.
2. **Building a Resilient and Integrated Health System:** Developing a decentralized healthcare system characterized by high quality, transparency, fairness, and accountability. This objective contributes to both leadership and governance and service delivery.
3. **Securing Diversified and Sustainable Funding Sources for Healthcare:** This objective directly addresses the health financing building block by identifying and securing diverse funding to ensure continuous quality service delivery.
4. **Nurturing Qualified National Talents and Promoting Scientific Research and Health Innovation:** This objective aligns with the health workforce and health information systems blocks by focusing on building national capabilities in research and innovation.
5. **Technology-driven Healthcare:** This objective focuses on developing sophisticated medical technology systems for high-quality preventive and clinical care, a core component of strengthening health information systems.

### 3.2. Context and Key Challenges

While Oman’s health system boasts strengths such as universal access for citizens and a strong public health infrastructure, a critical challenge is the inherent fragmentation of the health system itself [12,13]. The system is divided into separate service delivery networks, including the MoH system and the Medical City for military and security services, which operate with different governance structures, financing models, and service packages. This fragmentation leads to inefficient resource use, variable results, and creates a system that may not effectively and efficiently address the population’s health needs. A recent rapid performance assessment highlighted the need to unify health system planning and governance within the MoH to cover all service provision uniformly [13].

This challenge is compounded by the epidemiological transition, where the success in controlling communicable diseases has inadvertently accelerated the NCD burden, creating a “double burden” of disease that requires a reorientation of the healthcare system toward chronic disease prevention and management. The escalating prevalence of NCDs remains a primary concern, accounting for a majority of deaths and imposing a significant economic strain. This burden is quantified by the fact that NCDs cost Oman approximately OMR 1.1 billion annually in 2019, making the economic case for comprehensive policy intervention explicit. Despite the acknowledgment of this substantial cost, a critical gap exists in the country’s policy response to NCDs [14]. A legislative review noted that while numerous policies exist, many are ministerial regulations rather than comprehensive laws, particularly concerning food and physical activity promotion [15]. This creates a critical policy challenge for resource allocation and health system reorientation. A key trade-off is balancing the continuous need for investment in a strong communicable disease response with the urgent demand to shift resources toward effective NCD prevention and management strategies. The effectiveness of this shift is hindered by a lack of up-to-date data on NCD risk factors and an inadequate focus on population-based screening programs, which are often passive and miss a significant portion of the population.

### 3.3. Analysis of Achievements and Gaps by WHO Building Blocks

#### 3.3.1. Leadership and Governance

Leadership and Governance Oman’s move toward integrated governance and cross-institutional coordination represents a key policy intervention to overcome historical system fragmentation. A key manifestation of this change is the formation of the Committee for Managing and Regulating Healthcare, mandated to approve unified strategies and policies across all health institutions. Furthermore, the National Health Policy (launched in April 2025) serves as the comprehensive strategic framework, explicitly adopting the Health in All Policies (HiAP) approach [16]. The trade-off in this ambitious integration is the inevitable resistance to change from various stakeholders, and the systematic implementation of the HiAP approach remains challenging as multisectoral collaboration is often ad hoc and not fully institutionalized [17].

#### 3.3.2. Health Financing and Public–Private Partnerships (PPPs)

Oman is making tangible progress in the health financing building block by diversifying its funding sources. The initial success of this strategy is empirically demonstrated by the substantial capital attraction, with health sector projects totaling approximately OMR 506.6 million in 2023 [18]. PPPs are actively being pursued, exemplified by a project to manage a rehabilitation center in Sohar. In addition to these efforts, the Health Endowment Foundation (Athar) plays a pivotal role in this framework, connecting community initiatives with private sector resources to support the health sector. Nevertheless, successful PPP implementation requires meticulous governance frameworks to ensure equitable access and transparently manage financial risks.

While Oman’s shift towards Public–Private Partnerships (PPPs) and the new Social Protection Law for expatriates is a strategic move to foster a more inclusive framework, it introduces complex policy challenges and potential inequities. A key issue is the fragmentation of the health system, which creates parallel public and private service-delivery networks. This fragmentation, combined with a lack of a defined basic benefit package, creates a two-tiered system where many expatriates are solely reliant on private insurance or out-of-pocket (OOP) payments. The recent delay in implementing mandatory insurance for non-Omani workers under Royal Decree 52/2023, coupled with an estimated 70% of expatriates lacking insurance, highlights a significant gap between policy and practice that disproportionately affects vulnerable, low-wage migrant workers. This failure to close the coverage gap forces financial risk onto one of the most socially vulnerable populations and represents a critical barrier to achieving the social justice principles central to universal health coverage (UHC). The reliance on OOP payments by this group is not just a financial risk; it exacerbates pre-existing health disparities and creates a structural inequity where access to specialty care is conditional on employment status, thereby compromising the “Health in All Policies” (HiAP) commitment. This situation can also lead to disparities in access and financial protection, as private facilities are often concentrated in urban areas, creating a geographic barrier for a large segment of the population. Balancing the financial sustainability of the health system with the moral imperative to protect the health of all residents remains the most profound equity challenge for Vision 2040 financing reforms.

### 3.4. Strategic Efficiency and Governance Reforms

Oman’s strategic plan is actively working to improve the operational efficiency and cost-effectiveness of its healthcare system. This includes reducing unnecessary clinical procedures, which exhibit significant variation (e.g., some health centers perform up to 1189 complementary studies per 1000 visits). Crucially, a recent health efficiency report quantifies the potential savings, estimating that aligning all facilities with a median cost could result in a total efficiency gain of approximately 39 million OR across the health system [18]. Furthermore, an analysis using the Pabon Lasso model, a widely used method for assessing hospital efficiency, revealed that most of Oman’s hospitals have low bed occupancy rates, indicating significant excess capacity [18]. To address this, a reform agenda proposes a new needs-based formula for resource allocation that would consider the population served, their health needs, and the services provided, moving away from historical trends. The high number of outpatient visits in Oman (5.8 per person per year) compared to countries with high health outcomes like the Nordic countries (3.8 per year) suggests inefficiencies such as “doctor shopping” and weak referral processes [18]. In response, the government is committed to strengthening clinical pathways to standardize care and reduce these variations, which is expected to lead to significant cost savings.

## 4. Health Workforce

Progress in the health workforce building block is evidenced by the active implementation of the ‘Cadre Development and Capability Building Program’. This initiative focuses on national human resources, equitable distribution, and professional qualification, and has overseen over 1000 fellowships abroad from 2009–2024. The success of nationalization efforts is quantified by the achievement of high Omanization rates, reaching 95% for pharmacists and 96% for dentists. The Oman Medical Specialty Board (OMSB) plays a pivotal role in elevating medical professions, achieving international accreditation (ACGME-I) in 2023 and 2024. A significant trade-off remains in balancing the rapid “Omanization” of the health workforce with the need to retain a sufficient number of highly specialized expatriate staff to maintain service quality.

### 4.1. Health Information Systems and Digital Transformation

Oman’s experience provides a valuable case study in leveraging digital transformation to strengthen the health information systems building block. The implementation of technologies such as the updated ‘Al-Shifa’ App and AI-powered diagnostics demonstrates a proactive strategy to improve access, efficiency, and quality of care. However, while significant investments in the Al-Shifa system are a major achievement, independent assessments highlight persistent challenges in achieving a fully integrated and data-driven system [19]. This is evident between public providers like the MoH, Sultan Qaboos University Hospital, and military hospitals, which use different software platforms, creating a barrier to a truly integrated national record. This digital fragmentation also introduces new and complex risks [20]. A key concern for systems like Al-Shifa is cybersecurity, as the system’s fragmentation across different provider networks can create vulnerabilities that compromise patient data privacy and confidentiality [20]. Furthermore, the increasing use of artificial intelligence (AI) in diagnostics raises potential ethical concerns about algorithmic bias. The challenge of interoperability is compounded by the lack of a unified national health data exchange framework, a governance gap that countries like Estonia and Singapore have successfully overcome by implementing central, vendor-neutral data-sharing policies [21]. To mitigate the rising cybersecurity threat, Vision 2040 requires the urgent establishment of a centralized digital health authority focused on data governance, security protocols, and enforcing clear accountability across all disparate provider networks. Crucially, addressing the potential for algorithmic bias necessitates proactive ethical frameworks to ensure AI models are trained on representative data, protecting vulnerable populations from diagnostic inequity [22,23]. Successfully navigating these risks requires robust data governance, clear ethical frameworks, and continuous efforts to bridge the digital literacy gap among both healthcare providers and the general population to ensure that technology serves to enhance, not hinder, equitable care.

### 4.2. Service Delivery and Access to Essential Medicines

Oman has recorded notable progress in the service delivery building block by expanding its health infrastructure and improving patient care under Vision 2040. By the end of 2024, Oman had an extensive network of 192 health centers and 52 hospitals, with several new institutions and expansions underway. The Omani System for Accreditation of Health Institutions was launched in 2024, a strategic step to raise the quality of health services and ensure patient safety. Nevertheless, the trade-off here is balancing rapid infrastructure expansion with the meticulous, often slow, process of building a strong culture of quality assurance and patient safety.

## 5. Performance and Outcomes

The MoH’s progress toward achieving its Vision 2040 objectives is rigorously measured through a set of strategic performance indicators, which are subject to continuous monitoring and adjustment by the Oman Vision 2040 Implementation Follow-up Unit. This evaluation process assesses the overall performance of the health system across its various building blocks, with indicators flexible enough to keep pace with evolving local, regional, and global changes.

### 5.1. Legatum Prosperity Index-Health Pillar

Oman has demonstrated significant progress on the Legatum Prosperity Index, improving its international ranking by six positions since 2019 to reach 55th globally out of 167 countries, and ranking 6th out of 19 countries in the Middle East and North Africa [24]. This index measures physical and mental health, healthcare access, coverage, effectiveness, and health practices. Key achievements in 2024 and early 2025 contributing to this performance include the active application of the “Patient Safety Framework” in several hospitals and health centers and a significant improvement in the “Aman” incident reporting system, which recorded over 20,000 incidents in 2024. Additionally, several hospitals achieved international accreditation, and mental health services were integrated into primary healthcare centers.

A key challenge evidenced by this performance is the persistent gender disparity in NCD risk factors, with adult obesity prevalence reaching 23.2% for men and 38.8% for women in 2022 [25]. This trend underscores the need for more targeted, gender-sensitive public health interventions. Furthermore, while mental health services have been integrated into primary healthcare centers, there is a documented lack of data on population satisfaction and perceived needs, making it difficult to assess the system’s responsiveness. The absence of regular, representative population surveys on health behaviors and unmet needs represents a critical gap that hinders evidence-based policymaking and prevents a comprehensive understanding of the full impact of these health reforms.

### 5.2. Healthy Life Expectancy at Birth (HALE)

This indicator measures the average number of years an individual is expected to live in full health. The latest reading in 2021 showed an impact from the COVID-19 pandemic, with the average HALE decreasing to 63.2 years compared to 64.7 years in 2019 [26]. The MoH aims to reach 70 years by 2040 [1]. Key achievements in 2024 and early 2025 related to this indicator include the maintenance of a high immunization coverage rate, exceeding 99% for essential childhood vaccinations, and the expansion of the newborn screening program to include 26 diseases in 2024. In April 2025, Oman was declared free of measles and rubella for the fifth consecutive year. Furthermore, in early 2025, the National Survey for NCDs was launched to provide data for future planning, and has launched a national project for Diabetic Retinopathy (DR) screening using AI technologies, equipping 25 healthcare institutions to facilitate early detection and reduce vision loss among diabetic patients. As part of a free program in 2024, blood glucose monitoring sensors and insulin pumps were also distributed to Omani children with Type 1 diabetes.

### 5.3. Community Satisfaction Rate with Healthcare Services

**Figure 2:**
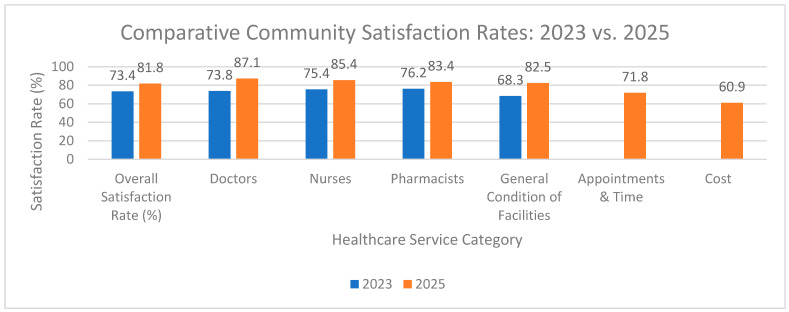
Comparative Community Satisfaction Rates: 2023 vs. 2025. The overall notable increase in satisfaction (from 73.4% to 81.8%) is evident across all areas; however, the improvement is not uniform, with disaggregated data revealing that the highest satisfaction rates remain concentrated in urban governorates, while rural regions show persistent gaps in areas like waiting times.

A key performance indicator under Oman Vision 2040 is the Community Satisfaction Survey with Healthcare Services. The latest report, published in June 2025, reveals an overall average satisfaction rate of 81.8% in 2025, marking a notable increase from 73.4% in 2023 [27]. The survey, which assesses satisfaction with areas such as doctors, nurses, and pharmacists, also introduced new metrics on satisfaction with appointments, time, and cost. Key initiatives contributing to this rise include promoting health awareness through over 114,000 diverse initiatives and partnerships in 2024, and the expansion of the Healthy Cities initiative, which now includes 50 recognized cities, villages, and islands. As illustrated in Figure 2, the data from the surveys shows a consistent upward trend in satisfaction across all key areas.

However, despite these positive indicators, the healthcare system continues to face challenges in ensuring equitable access and consistent quality, particularly between urban and rural areas. While the government’s strategic focus on decentralization aims to empower regional governance, a notable disparity in patient perception and satisfaction persists. The Community Satisfaction Survey reveals varying levels of satisfaction across the governorates. For example, satisfaction with appointments and waiting times varied, with Musandam Governorate reporting 76.9% compared to Al Dhahirah at 67.6% [27]. These findings suggest that potential root causes, such as differences in staffing ratios or access to advanced technology, may be more pronounced in less urbanized areas. To fully address these gaps, future research should critically analyze disaggregated data from the Community Satisfaction Survey by geographic location and other socioeconomic factors to provide a more precise, evidence-based measure of these disparities and inform targeted policy interventions.

According to the Arab Region SDG Index and Dashboards Report, Oman is among the top seven countries in the region that have completed two-thirds of the journey toward achieving the SDGs [28]. This positions Oman as a leader, but a more detailed comparison of key health indicators with other GCC nations reveals areas for further strategic focus. While Oman’s maternal mortality ratio and life expectancy are commendable, some GCC peers, have achieved even better outcomes. This highlights the need for continued investment in targeted interventions to further improve these metrics. Table 2 provides a comparative overview of key health-related SDG indicators, specifically Healthy Life Expectancy (HALE) and the Legatum Health Pillar Rank, serving as a basis for further research into the specific policy choices that drive performance in each country.

## 6. Generalizable Insights and Lessons for Global Health Policy

The experience of Oman’s health sector transformation under Vision 2040 offers a valuable blueprint for other nations navigating similar health system reforms. By detailing the country’s comprehensive approach, this study serves as a case model for policy interventions and implementation mechanisms that can provide generalizable insights of international interest.

### 6.1. Strategic Vision and Holistic Planning (HiAP)

Oman’s commitment to the HiAP approach, enshrined in its National Health Policy, underscores a crucial principle for addressing complex public health issues like NCDs. This model moves beyond traditional healthcare delivery to integrate health considerations into all policy-making, fostering shared responsibility across government, community, and the private sector. Nations seeking to tackle similar challenges can learn from Oman’s institutionalization of HiAP to ensure health is a collective responsibility and not solely confined to the health sector.

### 6.2. Integrated Governance and Implementation Mechanisms

The strategic shift from a historically fragmented healthcare system to a unified, integrated model with a central follow-up unit is a key policy lesson. Oman’s establishment of the Committee for Managing and Regulating Healthcare demonstrates a robust implementation mechanism for standardizing practices and optimizing resource allocation across diverse health entities. This strategic move provides a scalable model for countries aiming to overcome governance challenges and streamline their own health systems.

### 6.3. Innovative and Sustainable Health Financing

Oman’s efforts to diversify healthcare funding through PPPs and the activation of a national health insurance project address a critical global challenge of long-term financial sustainability. For nations grappling with growing healthcare demands and a reliance on volatile revenue sources, Oman’s strategic approach, including the role of the Health Endowment Foundation (Athar), offers a model for alleviating the state budget burden and attracting private investment.

### 6.4. Digital Transformation as a Policy Lever

Oman demonstrates how proactive digital health strategies can serve as powerful policy interventions to enhance access, efficiency, and quality of care. The rapid adoption of AI for diagnostics and the development of the “National Center for Virtual Health” project provide a model for other countries seeking to integrate technology to improve preventive and clinical services, particularly for managing chronic diseases.

### 6.5. Human Capital Development and Professional Standards

The “Cadre Development and Capability Building Program” and the role of the OMSB highlight the importance of investing in a highly skilled and resilient workforce. Oman’s strategy for training, equitable distribution, and retaining national healthcare professionals offers a blueprint for other nations aiming to build a capable workforce to meet future demands.

### 6.6. Performance Measurement and Adaptability

Oman’s commitment to data-driven decision-making, as evidenced by its use of key indicators like the Legatum Prosperity Index and HALE, demonstrates an adaptive governance approach. The flexibility of its monitoring system, managed by the Oman Vision 2040 Implementation Follow-up Unit, provides a valuable lesson for other nations on how to ensure accountability and make agile responses to evolving health landscapes.

## 7. Limitations of the Review

This review provides a comprehensive narrative synthesis of Oman’s health system transformation under Vision 2040; however, it is important to acknowledge several key limitations. First, this is a single-country case study, which, while providing rich contextual detail, may limit the direct generalizability of its findings to nations with different political, economic, or social contexts. While our analysis attempts to highlight broader policy lessons, the specific successes and challenges in Oman are unique to its national setting. Second, a primary limitation is the review’s reliance on official government reports and documents. While these are authoritative sources, they may be subject to a degree of institutional bias that presents a more favorable or incomplete narrative of progress. Our analysis attempts to mitigate this by critically synthesizing these findings and presenting them alongside reported challenges to provide a more balanced perspective. Third, the paper is a descriptive narrative synthesis rather than a quantitative or qualitative outcomes evaluation. It does not establish a causal link between the policies and the reported health outcomes. For instance, while community satisfaction rates have improved, the review cannot definitively prove that the new policies were the sole cause of this improvement. Finally, the review’s time horizon focuses on the period from 2023 to early 2025. While this provides a timely snapshot of recent progress, the long-term impact of Vision 2040’s health policies and interventions cannot be fully assessed at this stage.

## 8. Conclusions: A Strategic Path Forward for Oman’s Healthcare

Oman is strategically transforming its healthcare system in alignment with Vision 2040, aiming to build an internationally benchmarked model that can effectively manage the growing burden of NCDs. The Sultanate’s approach is comprehensive, encompassing the decentralization of services, empowerment of national cadres, integration of new technologies, and a focus on sustainable financing. These efforts are underpinned by a strong emphasis on the HiAP framework, which promotes a whole-of-society approach to health.

While significant progress has been made, persistent challenges remain, including the escalating prevalence of NCDs, the need for long-term financial sustainability, and the complexities of digital integration and PPPs. Successfully navigating these issues will require continuous adaptation and a commitment to evidence-informed policymaking to ensure equitable access and optimal resource allocation.

To achieve its ambitious goals, Oman’s healthcare strategy must be guided by robust, data-driven policymaking and concrete action. We recommend three strategic imperatives for the next phase of Vision 2040: First, to overcome system fragmentation and cybersecurity risks, policymakers should immediately establish a unified, vendor-neutral National Health Data Exchange Framework to ensure seamless data interoperability across all public health entities. Second, to guarantee digital equity and fair distribution of technological benefits, the system must introduce a Digital Equity Monitoring Index to regularly assess disparities in access to AI-powered diagnostics and telehealth services between urban and remote regions. Finally, to address the NCD burden more effectively, a mandate should be issued establishing Governorate-level HiAP Steering Committees with binding representation from non-health sectors (e.g., Education, Municipality) to institutionalize prevention and health promotion efforts. Aligning with global calls for evidence-based reform, future research should also prioritize longitudinal studies evaluating the long-term equity implications of new financing models and rigorously assessing the measurable impact of the Health in All Policies (HiAP) framework on demonstrably reducing the NCD burden. By prioritizing these imperatives, Oman can optimize resource allocation and serve as a model for other nations undergoing similar healthcare transformations.

## Figures and Tables

**Table 1:** Summary of Literature Retrieval and Selection Process.

Stage	Action	Count
Identification	Total records retrieved from database searches and organizational websites	500
	Records screened by title and abstract (after duplicate removal)	450
Eligibility	Full-text documents assessed for eligibility	450
	Documents excluded (lack of empirical data, irrelevance, duplication)	420
Inclusion	Total sources included in the final narrative synthesis	30

**Table 2:** Comparative Health-Related Indicators: Oman vs. GCC Peers and Global Benchmarks.

Country/Group	HALE (Years, 2022)	Legatum Health Pillar Rank (2023)	Policy Lessons
**Oman**	64.7	55	Focus on chronic disease prevention to increase full health years.
**UAE**	67.2	49	High performance indicates effective governance and investment in quality.
**Qatar**	70.1	40	Benchmark for achieving OECD-level outcomes in the region.
**Saudi Arabia**	64	45	Recent comprehensive policy reforms may accelerate future ranking gains.
**Bahrain**	65.9	50	Demonstrates balanced outcomes relative to regional peers.
**Kuwait**	66.7	52	Strong HALE suggests effective control over non-fatal morbidity.
**Middle East/North Africa**	63.6	-	Regional average defines the base challenge for policy intervention.
**Global Average**	61.9	-	-
**OECD Members (Average)**	70.2	-	Goal standard for high-income health system efficiency.

**Source:** World Health Organization (HALE, Ref. [25]) and Legatum Institute (Legatum Health Pillar Rank, Ref. [23]). **Takeaway:** While Oman is highly ranked, its HALE score remains below top GCC peers and the OECD average, indicating a need for continued focus on primary prevention to sustain full health into later years.

## Data Availability

The data supporting the findings of this narrative review and policy analysis are derived from official public documents and reports, including Oman Vision 2040 documents, Ministry of Health reports, and publications from international organizations (e.g., WHO, UNICEF). These source materials are cited within the manuscript’s reference list. No new datasets were generated or analyzed for this study.

## Abbreviations

The following abbreviations are used in this manuscript:

HiAP	Health in All Policies
NCD	Non-Communicable Diseases
SDGs	global Sustainable Development Goals
HALE	Healthy Life Expectancy at Birth
WHO	World Health Organization’s
MoH	Ministry of Health
UNICEF	United Nations Children Fund
DR	Diabetic Retinopathy
PPPs	Public–Private Partnerships
OMSB	Oman Medical Specialty Board
